# Identification and Validation of SNP-Containing Genes With Prognostic Value in Gastric Cancer *via* Integrated Bioinformatics Analysis

**DOI:** 10.3389/fonc.2021.564296

**Published:** 2021-04-27

**Authors:** Hui Li, Jing Guo, Guang Cheng, Yucheng Wei, Shihai Liu, Yaoyue Qi, Gongjun Wang, Ruoxi Xiao, Weiwei Qi, Wensheng Qiu

**Affiliations:** ^1^ Department of Medcine, Qingdao University, Qingdao, China; ^2^ Department of Oncology, Affiliated Hospital of Qingdao University, Qingdao, China; ^3^ Central Laboratory, Affiliated Hospital of Qingdao University, Qingdao, China

**Keywords:** bioinformatics analysis, biomarkers, gastric cancer, prognosis, single nucleotide polymorphisms

## Abstract

**Background:**

Gastric cancer is one of the most common malignancies worldwide. Although the diagnosis and treatment of this disease have substantially improved in recent years, the five-year survival rate of gastric cancer is still low due to local recurrence and distant metastasis. An in-depth study of the molecular pathogenesis of gastric cancer and related prognostic markers will help improve the quality of life and prognosis of patients with this disease. The purpose of this study was to identify and verify key SNPs in genes with prognostic value for gastric cancer.

**Methods:**

SNP-related data from gastric cancer patients were obtained from The Cancer Genome Atlas (TCGA) database, and the functions and pathways of the mutated genes were analyzed using DAVID software. A protein-protein interaction (PPI) network was constructed using the STRING database and visualized by Cytoscape software, and molecular complex detection (MCODE) was used to screen the PPI network to extract important mutated genes. Ten hub genes were identified using cytoHubba, and the expression levels and the prognostic value of the central genes were determined by UALCAN and Kaplan-Meier Plotter. Finally, quantitative PCR and Western blotting were used to verify the expression of the hub genes in gastric cancer cells.

**Results:**

From the database, 945 genes with mutations in more than 25 samples were identified. The PPI network had 360 nodes and 1616 edges. Finally, cytoHubba identified six key genes (TP53, HRAS, BRCA1, PIK3CA, AKT1, and SMARCA4), and their expression levels were closely related to the survival rate of gastric cancer patients.

**Conclusion:**

Our results indicate that TP53, HRAS, BRCA1, PIK3CA, AKT1, and SMARCA4 may be key genes for the development and prognosis of gastric cancer. Our research provides an important bioinformatics foundation and related theoretical foundation for further exploring the molecular pathogenesis of gastric cancer and evaluating the prognosis of patients.

## Introduction

Gastric cancer is the fifth most common malignant tumor worldwide and the second leading cause of cancer-related death (1). Although the treatment strategies for gastric cancer have substantially improved in recent years, the mortality rate is still high due to various genetic mutations and abnormal signaling pathways underlying the progression of this disease (2). The occurrence and development of gastric cancer, as a complex disease, involves a series of genetic, epigenetic and phenotypic changes. Gene polymorphisms involved in multiple biological pathways have been identified as potential risk factors for gastric cancer (3). Given the high morbidity and mortality of gastric cancer, identification of its underlying molecular mechanism and genetic characteristics and elucidation of biological indicators for diagnosis and prognosis are essential for the personalized and precise treatment of gastric cancer patients.

Bioinformatics analysis based on high-throughput sequencing is an important method for exploring the molecular mechanism of tumor pathogenesis, identifying biomarkers that can be used for early diagnosis, and discovering therapeutic targets. Single nucleotide polymorphisms (SNPs) are nucleotide polymorphisms that are commonly found in the genome of an organism; among individuals of different species, single nucleotides in the same position of the genomic DNA sequence undergo substitution, insertion or deletion and other mutations, resulting in a single nucleotide change at this site (4). SNPs are generally considered to be the genetic basis of and potential cancer markers that lead to differences in the individual susceptibility to disease. Analysis of SNP-containing genes is important for the early diagnosis and individualized targeted treatment of cancer.

The Cancer Genome Atlas (TCGA) database can be used for high-throughput genomic analysis. To further explore the biological significance of SNP-containing genes in the diagnosis and prognosis of gastric cancer, we downloaded gastric cancer-related SNP data from TCGA database and used bioinformatics analyses, including mutation analysis, function and pathway enrichment analyses, protein-protein interaction (PPI) network analysis and related analyses. Our aim was to explore the mutated genes related to the diagnosis and prognosis of gastric cancer and to provide a scientific theoretical basis for personalized and precise treatment of gastric cancer.

## Materials and Methods

### Data Processing and Analysis

The TCGA data portal was terminated, and all TCGA data were transferred to the newly established genomic data sharing platform (https://gdc.cancer.gov/) (5). Since the original data on SNPs in TCGA have not yet been opened to the public, we downloaded the processed SNP-related data of gastric cancer and the original mRNA expression data. The mRNA data were collected from 413 samples (including 32 normal samples and 381 cancer samples). The mutated genes were obtained from the SNP data of the downloaded gastric cancer samples. The edgeR software package was used to integrate and standardize the downloaded mRNA raw data, and analysis was performed to obtain the differentially expressed genes and their expression levels. The mRNA data provided by TCGA are publicly available, and thus, no approval from the local ethics committee was required.

### Functional Enrichment and Pathway Analysis of Mutated Genes

To elucidate the dysfunction caused by these mutated genes, we used the DAVID (http://www.DAVID.org) (6) database to perform Gene Ontology (GO) and Kyoto Gene and Genome Encyclopedia (KEGG) analyses on genes from more than 25 mutated samples. As an open source platform, DAVID can be used to determine the association between target molecules. By selecting the GO term and the KEGG pathway and using P <0.05 as the cut-off value, we identified the molecular functions (MFs), biological processes (BPs), cellular components (CCs) and KEGG pathways of the enriched mutated genes.

### Construction of the PPI Network of the Mutated Genes and Gene Expression Analysis

The STRING database (http://string-db.org/) provides the significant PPIs (7). Cytoscape is used for visual exploration of interactive networks (8). In this study, the STRING database was used to perform PPI network analysis of the selected SNP-containing genes, and then, Cytoscape visualization was used, with a confidence score> 0.4 as the cut-off criterion. The Cytoscape plug-in cytoHubba (9) was used to identify the hub genes by finding the intersections of the first 30 genes from 12 topological analysis methods and then using molecular complex detection (MCODE) to establish the module of the PPI network, with a degree cutoff = 2, node score cutoff = 0.2, k‐core = 2, and max depth = 100.17 (10).

### Kaplan-Meier Survival Curve of the Mutated Genes and Screening of Prognostic Biomarkers

Kaplan-Meier Plotter (https://kmplot.com/analysis/) can use gene expression data to assess the survival rate of breast, lung, gastric and ovarian cancer patients. Recurrence-free survival (RFS) and overall survival (OS) data were downloaded from GEO (Affymetrix microarray only), EGA and TCGA. The main purpose of this tool is biomarker evaluation based on meta-analysis (11). Using the Kaplan-Meier chart, we evaluated the effects of the mutated genes on the prognosis of gastric cancer patients and finally identified genes that can be used as prognostic biomarkers for this disease.

### Hub Gene Verification Through UALCAN

UALCAN (http://ualcan.path.uab.edu/) is a web-based tool that can provide fast and customizable functions based on level 3 RNA-seq and clinical data of 31 cancer types from TCGA database (12). In this study, the UALCAN database was used to verify the expression of the central genes identified in the module between normal and cancer samples. We chose P <0.05 and fold change> 2 as the threshold.

### Analysis of Cancer Genomics Data Through cBioPortal

cBioPortal for Cancer Genomics (http://cbioportal.org) provides resources for visualizing and analyzing multidimensional cancer genomics data (13). In this study, based on mutations and changes in the DNA copy number of the four selected subtypes of gastric cancer, we performed an analysis of the genomic changes in pivotal genes.

### Cell Culture and Antibodies

AGS, HGC27 and GES-1 cell lines were purchased from the Cell Bank of the Chinese Academy of Sciences and cultured in RPMI-1640 medium containing 10% fetal bovine serum (FBS). FBS and RPMI-1640 were purchased from Gibco (NY, USA). The cells were placed in an incubator at 37°C and a CO2 concentration of 5%. Antibodies against TP53 (#2527), BRCA1 (#14823), PIK3CA (#4255), AKT1 (#2938), and SMARCA4 (#49360) were purchased from Cell Signaling Technology (Beverly, MA, USA). Antibodies against HRAS (abs137096) and beta actin antibody (abs132001) were obtained from Absin (Shanghai, China). The secondary antibodies used in this study include goat anti-mouse IgG-HRP (abs20001) and goat anti-rabbit IgG-HRP (abs20002), both of which can be obtained from Absin.

### qPCR for Detection of the Expression Levels of the Hub Genes

According to the manufacturer’s instructions, total RNA was isolated from cells using TRIzol reagent (TaKaRa, Beijing, China) and reverse transcribed into cDNA using PrimeScript RT Master Mix reagent (TaKaRa, Beijing, China). Quantitative real-time PCR (qRT-PCR) was performed using the ABI 7500HT Fast real-time PCR system (Applied Biosystems, California, USA), and then, melting curve analysis was performed. The following cycling conditions were used: 95°C for five minutes, followed by 40 cycles of 95°C for 20 seconds and 60°C for 30 seconds. We used the 2-ΔΔCt method, with GAPDH as an internal control, to determine the average relative fold change in mRNA expression. The primers are shown in **Table 1**.

**Table 1:** The primer of hub genes.

Primer name	Sense	Antisense
TP53	GAGGTTGGCTCTGACTGTACC	TCCGTCCCAGTAGATTACCAC
HRAS	GACGTGCCTGTTGGACATC	CTTCACCCGTTTGATCTGCTC
BRCA1	GAAACCGTGCCAAAAGACTTC	CCAAGGTTAGAGAGTTGGACAC
PIK3CA	AGTAGGCAACCGTGAAGAAAAG	GAGGTGAATTGAGGTCCCTAAGA
AKT1	AGCGACGTGGCTATTGTGAAG	GCCATCATTCTTGAGGAGGAAGT
SMARCA4	GAAACAAGACGACTTTGTGACCT	CTTCACGGTTGCCTACTGGT

### Western Blot Analysis

The gastric cancer cells were inoculated into a 6 cm Petri dish, treated for 48 hours, scraped and collected. The cells were dissolved on ice in PMSF-containing RIPA buffer, and then, the mixture was centrifuged at 13,000 x g at 4°C for 5 minutes to remove the cell debris. The supernatant was collected, and the total protein concentration was determined using the BCA protein assay kit. Approximately 20 μg of protein was separated by 15% sodium dodecyl sulfate-polyacrylamide gel electrophoresis. The protein was wet transferred to a 0.22 μm polyvinylidene fluoride (PVDF) membrane using a constant current of 300 mA, blocked with 5% skim milk powder in TBST for 2 hours and incubated overnight with the appropriate primary antibody (1:1000). The next day, the membrane was washed 3 times with TBST for 10 minutes. At room temperature, the membrane was incubated with the HRP-conjugated secondary antibody (1:8000) for 2 hours and washed with TBST 3 times for 10 minutes each time. A chemiluminescence kit (Life Technologies, Shanghai, China) was used to observe the bound antibody under the Bio-Rad gel imager infrared imaging system (ChemiDoc XRS +).

### Statistical Methods

Data are means ± standard deviation. Two-tailed unpaired Student’s t tests were used to assess significance unless stated otherwise. P < 0.05 was deemed significant.

## Results

### Data Processing and Analysis

**Figure 1:**
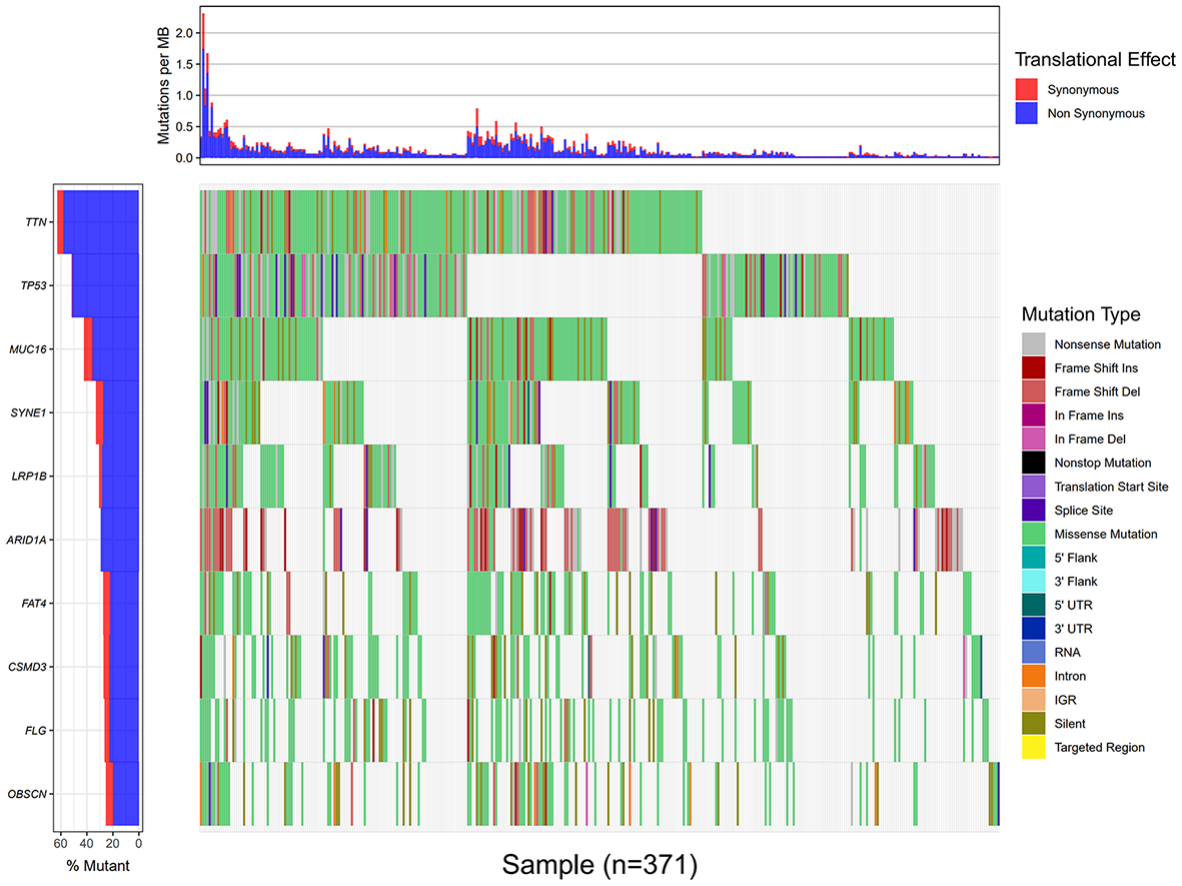
Data processing and analysis. A waterfall map of 10 genes that were mutated in more than 90 samples.

**Figure 2:**
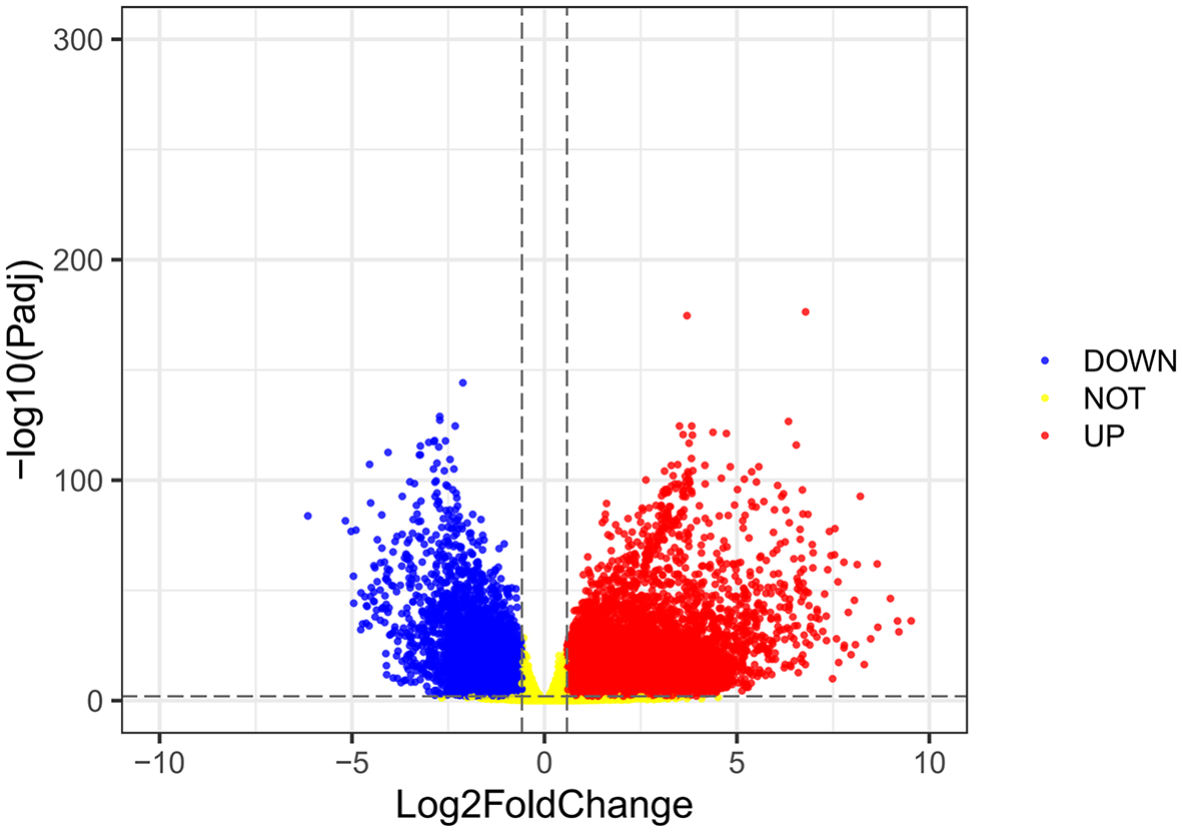
A volcanic map of differential gene expression. The red dot represents the upregulated mRNAs, and the green dot represents the downregulated mRNAs.

Using the VarScan method to extract germ/somatic cell mutation data of gastric cancer samples from the second-generation sequencing data of TCGA database as SNP data, we selected 945 genes with mutations in more than 25 samples. Among these genes, 96 genes were mutated in more than 50 samples (**Figure 1**). From TCGA database, 413 samples with gastric cancer gene expression data, including 32 normal tissue samples and 381 cancer tissue samples, were obtained. The patient characteristics are in **Supplementary Table 1**. The edgeR software package was used to analyze the differential expression between the gastric cancer and normal tissue samples (**Figure 2**), with |log FC|> 2 and P <0.01 as the cut-off criteria. We further analyzed the SNP-containing genes and differentially expressed genes in gastric cancer to explore the dysfunction caused by gene mutations and abnormal expression.

### Functional Enrichment and Pathway Analysis of the Mutated Genes

**Figure 3:**
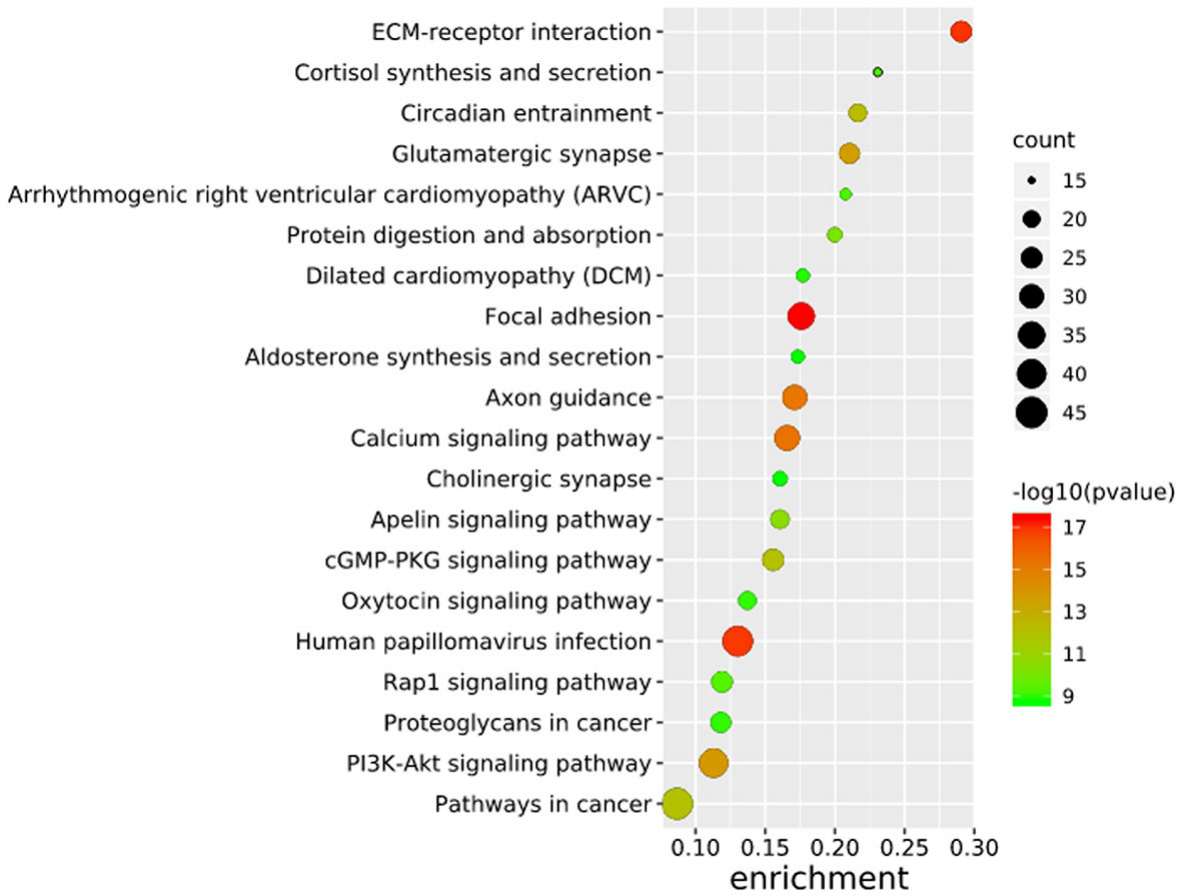
Pathways enrichment map of 945 mutant genes. The top 20 terms with the lowest P value were selected. Count: the number of enriched genes in each term.

To further elucidate the functions of the mutated genes in gastric cancer, we used DAVID online software to perform functional enrichment analysis and pathway analysis on the 945 genes with mutations in more than 25 samples. Functional enrichment analysis showed that in the BP categories, SNP-containing genes were mainly concentrated in homophilic cell adhesion *via* plasma membrane adhesion molecules, membrane depolarization during action potential and neuronal action potential. In the CC categories, mutated genes were mainly categorized in the plasma membrane, proteinaceous extracellular matrix, and voltage-gated sodium channel complex. In the MF categories, these genes were mainly enriched in calcium ion binding, extracellular matrix structural constituent and voltage-gated sodium channel activity (**Table 2**). Pathway enrichment analysis revealed the enrichment of the SNP-containing genes in many signaling pathways related to cancer, including the phosphatidylinositol 3 kinase (PI3K)/Akt signaling pathway, calcium signaling pathway, and cyclic guanosine monophosphate (cGMP)-PKG signaling pathway (**Figure 3**).

**Table 2:** Gene ontology analysis of 945 mutant genes in gastric cancer.

Category	Term	Count	*P* value
GOTERM_BP_DIRECT	homophilic cell adhesion *via* plasma membrane adhesion molecules	54	1.13E-35
	negative regulation of transcription from RNA polymerase II promoter	33	0.0075481
	intracellular signal transduction	26	0.0033163
	cell adhesion	23	2.98E-06
	membrane depolarization during action potential	18	8.42E-15
	axon guidance	17	1.11E-05
	heart development	16	2.29E-04
	cell migration	13	0.0085702
	neuronal action potential	12	0.0337519
	microtubule cytoskeleton organization	12	1.60E-09
GOTERM_MF_DIRET	GO:0005509~calcium ion binding	99	3.414E-26
	GO:0005524~ATP binding	99	1.153E-06
	GO:0008270~zinc ion binding	65	0.0263632
	GO:0003677~DNA binding	42	0.0361054
	GO:0003682~chromatin binding	25	0.0180564
	GO:0044212~transcription regulatory region DNA binding	18	9.495E-06
	GO:0005096~GTPase activator activity	18	0.0093759
	GO:0016887~ATPase activity	16	5.068E-05
	GO:0004842~ubiquitin-protein transferase activity	15	0.0201426
	GO:0004725~protein tyrosine phosphatase activity	14	9.479E-09
GOTERM_CC_DIRECT	integral component of membrane	197	0.0385191
	plasma membrane	133	2.329E-12
	integral component of plasma membrane	54	0.0021151
	proteinaceous extracellular matrix	32	6.187E-12
	cell surface	32	0.0006393
	focal adhesion	31	0.0002001
	dendrite	22	8.302E-07
	neuronal cell body	18	6.336E-05
	voltage-gated potassium channel complex	15	6.437E-07
	postsynaptic density	14	2.216E-07

Top 10 terms were selected according to count and P value <0.05. Count: the number of enriched genes in each term.

### Construction of the PPI Network for the Mutated Genes

**Figure 4:**
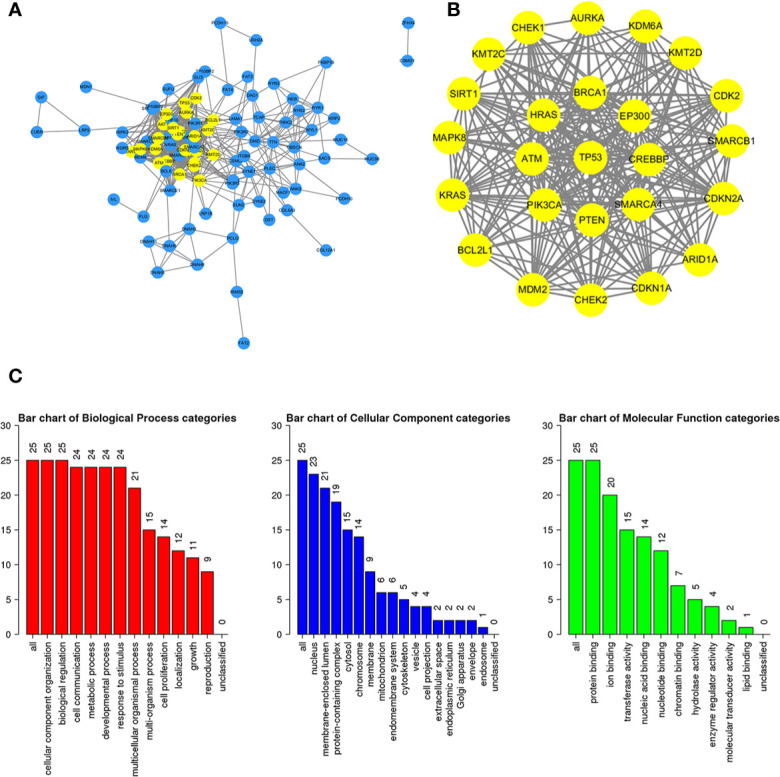
Construction of the PPI network for the mutated genes and analysis of important modules. **(A)** Cytoscape was used to construct a PPI network of 945 mutant genes, including 360 nodes and 1616 edges. **(B)** MCODE was used to obtain an important module with 25 nodes and 245 edges from the PPI network. **(C)** The functional enrichment histogram of important modules. Each biological process, cellular component and molecular function category is represented by a red, blue and green bar, respectively. The height represents the number of IDs in the user list and in the category.

**Figure 5:**
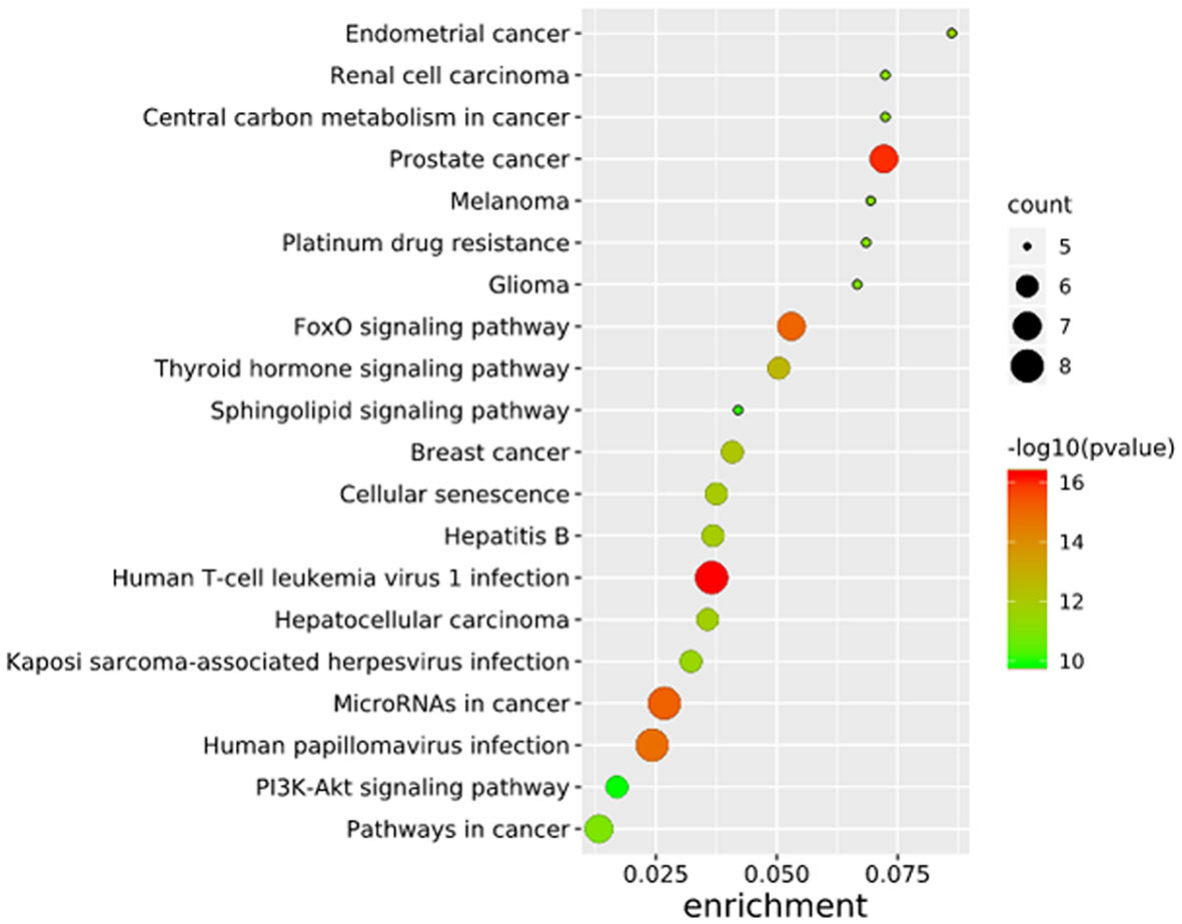
Pathways enrichment map of important modules.

To further study the potential relationships between the mutated genes, we used the STRING online database to mine the interactions among these genes. Cytoscape software was used to visualize the complex PPI network, which included 360 nodes and 1616 edges (**Figure 4A**). MCODE was used to obtain the important modules from the PPI network, including 25 nodes and 245 edges (**Figure 4B**). Functional and KEGG pathway enrichment analyses showed that the BP categories of important modules mainly included CC organization, biological regulation and cell communication; the CC categories included nucleus, membrane-enclosed lumen and protein-containing complex; and the MF categories mainly included protein-binding, ion binding and transferase activity (**Figure 4C**). KEGG pathway enrichment analysis showed that important module genes were mainly enriched in the FoxO signaling pathway and thyroid hormone signaling pathway (**Figure 5**).

### Screening and Survival Analysis of Pivotal Genes

**Figure 6:**
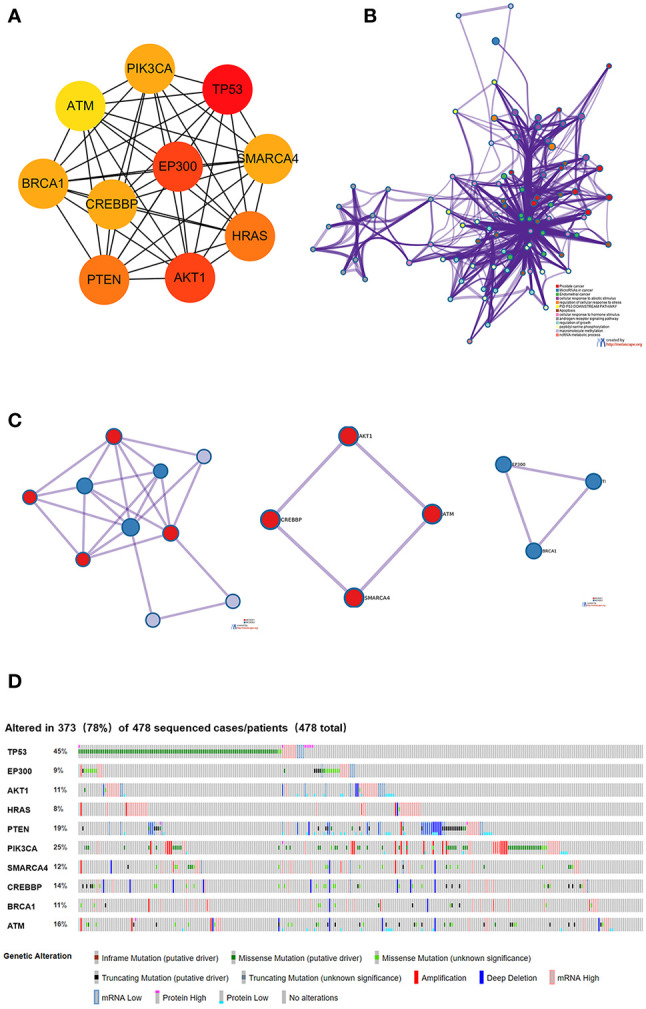
Selection and analysis of pivotal genes. **(A)** Identification of the 10 most important central genes using the Cytoscape software plug-in cytoHubba. **(B)** Metascape tools were used to analyze the pathway and biological process enrichment of hub genes. **(C)** Protein-protein interaction network and MCODE components identified in the gene lists. **(D)** Graphic analysis of the genetic alteration of key genes.

**Figure 7:**
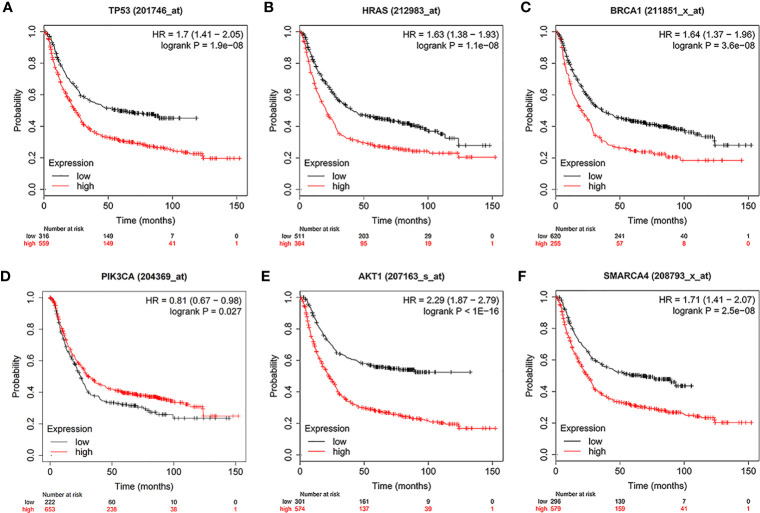
Univariate survival analysis of the key genes using Kaplan-Meier curves. **(A)** TP300. **(B)** HRAS. **(C)** BRCA1. **(D)** PIK3CA. **(E)** AKT1. **(F)** SMARCA4.

Using the intersection of the first 30 genes in cytoHubba’s 12 algorithms, we identified 10 key genes: TP53, EP300, AKT1, HRAS, PTEN, PIK3CA, SMARCA4, CREBBP, BRCA1 and ATM (**Figure 6A**). Metascape tools were used to analyze the pathway and biological process enrichment of hub genes. We observed that key genes are enriched in the PID P53 downstream pathway, apoptosis, regulation cellular response to stress, etc (**Figures 6B, C**). The cBioPortal online platform provided a graphic analysis of the genetic variation of the hub genes. As shown in the figure, 10 key SNP-containing genes all showed a high mutation rate in gastric cancer, with a rate of genome change ranging from 8% to 45% (**Figure 6D**). To determine whether the selected hub genes have clinical correlations, we used Kaplan-Meier curves to analyze the univariate survival of these genes and found that the expression of TP53, HRAS, BRCA1, PIK3CA, AKT1 and SMARCA4 was correlated with prognosis (**Figure 7**). Thus, these genes can be used as prognostic indicators of gastric cancer.

### Hub Gene Verification Through UALCAN

**Figure 8:**
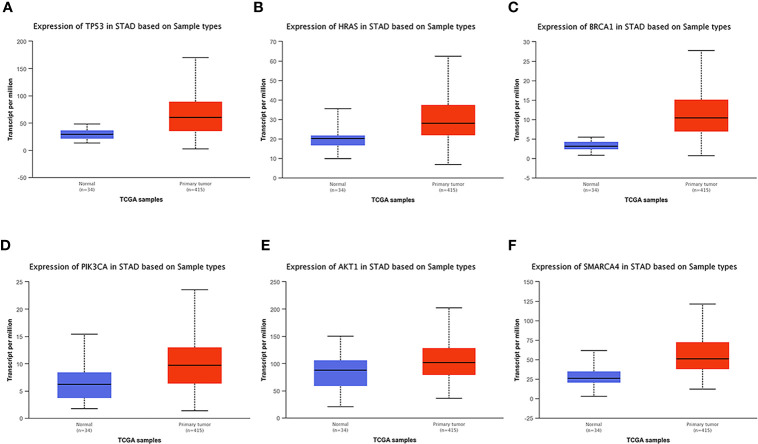
Analysis of the expression of key mutated genes. Six key genes are highly expressed in gastric cancer tissues compared with normal tissues. **(A)** TP300. **(B)** HRAS. **(C)** BRCA1. **(D)** PIK3CA. **(E)** AKT1. **(F)** SMARCA4.

UALCAN, an online tool with data from TCGA and GTEx, was used to verify the expression of these key genes in gastric cancer. In this study, according to the RNA sequence data from TCGA database, the mRNA expression levels of 6 genes were compared between the gastric tumor samples and the adjacent normal tissues. These six genes were found to be highly expressed at the transcriptional level in 415 gastric cancer tissues compared with 34 normal tissues (**Figure 8**). We chose P<0.05 and multiple change>2 as the threshold.

### Genomic Changes of the Hub Genes

**Figure 9:**
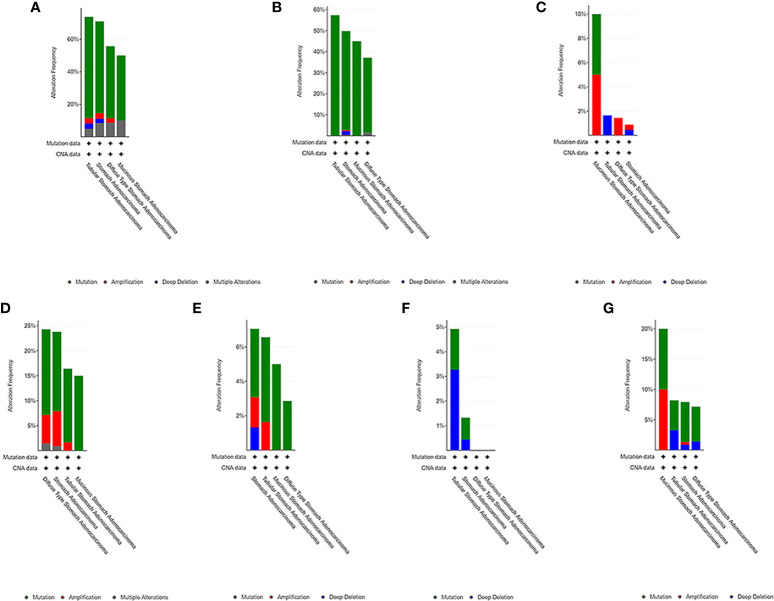
Genome-specific changes in the pivotal genes in 5 gastric cancer data sets. **(A)** All hub genes; **(B)** TP53; **(C)** HRAS; **(D)** BRCA1; **(E)** PIK3CA; **(F)** AKT1; **(G)** SMARCA4. Each row represents a gene, and each column represents a tumor sample. Red bars, gene amplifications. Blue bars, deletions. Green squares, missense mutations.

We used the cBioPortal tool to select 478 samples from TCGA database and explored the genome-specific changes of the hub gene. A summary analysis of cancer types showed that in the gastric cancer data set from TCGA, the proportion of the 6 genes changed from 12.66% to 51.90%, with the lowest to highest levels in mucinous stomach adenocarcinoma, diffuse type stomach adenocarcinoma, stomach adenocarcinoma, and tubular stomach adenocarcinoma (**Figure 9**).

### Gene Expression Levels of the Six Genes in Gastric Cancer

**Figure 10:**
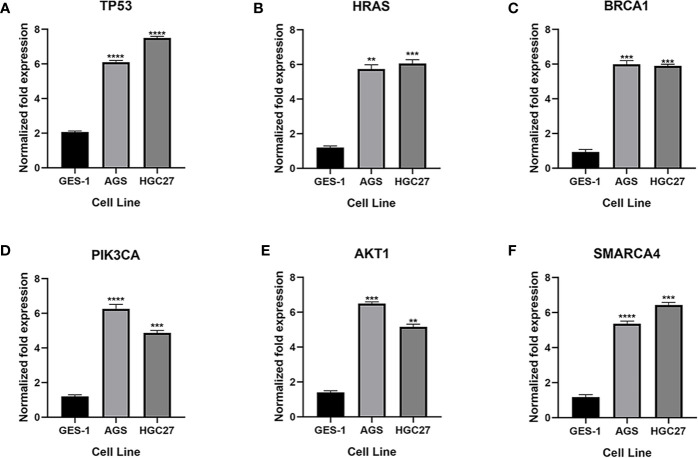
Expression of the key genes in gastric cancer cells. **(A-F)** qRT-PCR analysis of mRNA expression of key genes in GES-1, MGC803 and AGS cells. **P < 0.01, ***P < 0.001, and ****P < 0.0001.

**Figure 11:**
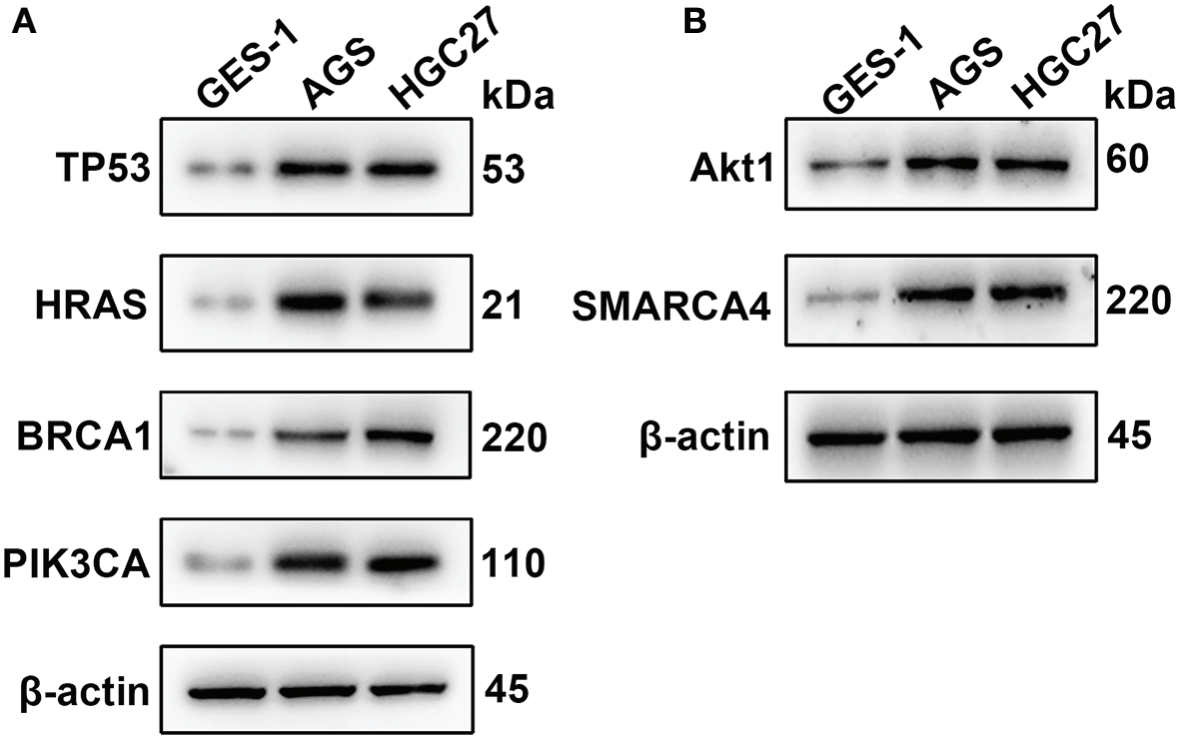
Western blotting analysis of the key genes; β-actin was used as a loading control. **(A)** Protein expression of TP53, HRAS, BRCA1 and PIK3CA. **(B)** Protein expression of AKT1 and SMARCA4.

qRT-PCR was used to analyze the expression of TP53, HRAS, BRCA1, PIK3CA, AKT1, and SMRACA4 in gastric cancer. The results showed that the expression of the hub genes in AGS and GES-1 cell lines was upregulated compared with that in GES-1 cell lines (**Figure 10**). Western blot results showed that the expression levels of the six genes in gastric cancer cells were significantly higher than those in normal cells (**Figure 11**).

## Discussion

Gastric cancer is a complex disease and the fifth most common malignant tumor worldwide; it is also the third leading cause of cancer-related death (14). To improve the quality of life and prognosis of patients and prolong their survival time, researchers must further clarify the molecular mechanism leading to malignant biological behavior of gastric cancer and identify prognostic markers that affect the development of this disease. According to previous reports, genetic polymorphisms will increase the risk of cancer and are considered to be indicators of poor prognosis in various cancers and potential carcinogenic markers. Therefore, bioinformatics analysis of SNP-containing mutant genes and selection of valuable genes can provide new tools to treat patients and predict prognosis in the clinic.

In this study, we conducted a series of bioinformatics analyses on gastric cancer-related data in TCGA database to screen and identify prognostic biomarkers related to SNP-associated expression. We conducted functional and pathway enrichment analyses of these genes and found that these genes are enriched in the nucleus and protein complex, mainly regulating multicellular BPs, developmental processes and metabolic processes. Pathway analysis showed that genes mutated in gastric cancer are mainly involved in the PI3K-AKT pathway, CGMP-PKG pathway, calcium signaling pathway, and many other cancer-related pathways. Functional and pathway enrichment analyses revealed the molecular mechanism of SNP-containing genes in the development of cancer.

Six mutated genes, TP53, AKT1, HRAS, PTEN, PIK3CA, SMARCA4 and BRCA1, which are closely related to the occurrence and development of gastric cancer, were screened. Survival analysis showed that the high expression of these six genes was associated with poor prognosis of gastric cancer. Using UALCAN online analysis, we found that the expression of the six genes in gastric cancer tissues was significantly higher than that in normal tissues. Subsequent cell experiments confirmed this result. In addition, we also used cBioPortal tools to study the genomic changes of the key genes in patients with gastric cancer from TCGA database. We found that there were five types of gastric cancer, and tubular gastric adenocarcinoma had the highest frequency of mutations in these genes. The rates of alteration of seven genes ranged from 12.66% to 51.90%.

TP53 (tumor protein p53) is the gene with the highest mutation frequency in gastric cancer (approximately 50%), and it is also the most commonly mutated gene in human cancer. This gene plays an important role in cell cycle arrest, cellular senescence, apoptosis, differentiation and metabolism (15). As a research hotspot in the field of tumor molecular biology, mutations in this gene are related to the poor prognosis of various cancers (16). Most TP53 mutations are missense mutations and gene deletions caused by substitution of single nucleotides, resulting in changes in the TP53 activity. Mutant p53 protein not only loses the antitumor effect of wild-type p53 protein but also increases tumor cell activity, invasion and metastasis and promotes the occurrence and progression of tumors (17). Previous studies have demonstrated the relationship between TP53 and gastric cancer. Ando et al. (18) studied clinical samples from 182 cases of gastric cancer and found that TP53-positive tumors had deeper invasion and more lymph node and liver metastasis than other tumors, and some genes (PICT1, RPL11) were involved in the progression of cancer through TP53 (19). TP53 mutations occur late in gastric carcinogenesis, contributing to the final transition to cancer (20). In addition, Jiang et al. found that TP53 mutation can inhibit tumor immunity in gastric cancer (21). According to the results of our analysis, the degree value of the TP53 gene is the highest among the 10 hub genes, and its increased expression is negatively correlated with the five-year survival rate of gastric cancer, which further confirms the validity of this study. Further exploration of TP53 mutant genotypes will help reveal the molecular mechanisms underlying the occurrence and development of gastric cancer.

HRAS is a member of the RAS gene family, which participates in the activation of RAS protein signal transduction. The RAS protein is a GDP/GTP-binding protein that mainly regulates proliferation, differentiation and senescence in wild-type cells (22). HRAS functions as an oncogene after activation, and activation commonly occurs through mutations (SNPs, insertions, translocations) and increased expression (23). Diseases related to HRAS include breast cancer, liver cancer, thyroid cancer, and bladder cancer (24–27). Ectopic expression of HRAS was shown to promote the proliferation, migration, invasion, angiogenesis and clone formation of gastric cancer cells (28). Our results suggest that HRAS may play an important role in the diagnosis and treatment of gastric cancer.

Breast cancer susceptibility gene (BRCA1) belongs a class of tumor suppressor genes with high penetrance that plays an important role in the response to DNA damage (including DNA double-stranded breaks) (29). The BRCA1 protein can bind to various proteins to regulate gene transcription, maintaining the integrity of the genome. BRCA1 gene mutation leads to DNA replication errors and mutations, which promote abnormal cell proliferation and lead to tumorigenesis (30). Increasing evidence shows that BRCA1 is widely associated with breast, ovarian and colon cancers (31, 32). Interestingly, the location of BRCA1 in gastric cancer cells was reported to be different, and different expression levels were observed. The expression of BRCA1 in the cytoplasm is downregulated, and the expression of BRCA1 in the nucleus is upregulated, which is related to the poor prognosis of advanced tumors (33). BRCA1 gene polymorphisms have also been associated with susceptibility to gastric cancer (34, 35). Our study showed that the expression of BRCA1 in gastric cancer was higher than that in normal samples, and high expression was associated with poor prognosis, which indicates that BRCA1 may play contrasting roles in different types of tumors, and the role of BRCA1 in gastric cancer should be further explored.

The PI3K-Akt signal transduction pathway plays an important role in tumorigenesis, development, treatment and prognosis (36). PI3K is a component of the PI3K signaling pathway, plays a key role in the regulation of cell proliferation, survival and adhesion and is often upregulated in human cancer (37). PIK3CA (phosphoinositide-3-kinase, catalytic, alpha gene) encodes the p110α subunit of PI3K. It plays an important role in tumor cell proliferation, differentiation, transport and metabolism (38). In addition, the PIK3CA pathway regulates angiogenesis and the immune response to cancer (39). PIK3CA mutations have been found in approximately 30% of human cancers (40), including breast cancer, ovarian cancer, colon cancer, and prostate cancer (41–44). In gastric cancer, high PIK3CA protein expression is closely related to tumor invasiveness, tumor phenotype and poor survival of patients (45). PIK3CA mutations were associated with high T stage, poor differentiation and microsatellite instability (46, 47). In our study, correlation analysis showed that the expression of PIK3CA in tumor tissues was significantly higher than that in normal tissues. However, OS analysis indicated theassociation of better prognosis of gastric cancer with highexpression of PIK3CA, suggesting that the role of PIK3CA ingastric cancer is worthy of further exploration.

AKT is a direct downstream target protein of PIK3. Increasing evidence shows that the activation of the AKT protein plays an important biological role in the development of cancer (48). AKT1 is one of the subtypes of AKT. Activated AKT1 phosphorylates many downstream substrates and participates in the regulation of cell growth, metabolism, proliferation, apoptosis and other processes (49). Petrini et al. found that patients with overexpression of AKT1 in gastric cancer had a poor prognosis, suggesting that AKT1 can be used as a poor prognostic marker for gastric cancer (50). Ghatak et al. found that AKT1 mutation was associated with an abnormal cell cycle in gastric cancer (51). Pathway analysis showed that AKT1 is enriched in PI3K-AKT, MAPK and several other pathways closely related to cancer, which indicates that the gene encoding AKT1 has an important biological function in the development of cancer.

ATP-dependent chromatin remodeling plays an important role in the occurrence and development of cancer, participating in almost all aspects of DNA metabolism, such as transcription, recombination, DNA repair and DNA replication (52). The SWI/SNF complex (BAF complex) was the first discovered mechanism of chromatin remodeling. Proteins encoded by SMARCA4 (also known as BGR1) are members of the SWI/SNF family, have helicase and ATP enzyme activities, and regulate gene transcription by changing the structure of chromatin (53). SMARCA4 is generally considered a tumor suppressor gene (54). However, some recent reports have demonstrated that SMARCA4 plays an important role in cell survival and proliferation in some types of cancer (55, 56). Martinez et al. found that SMARCA4 was highly expressed in 11 kinds of tumor tissues, including gastric cancer tissue, and was related to poor prognosis (57), which reflects the dual role of SMARCA4 in cancer. Previous studies have identified several genes as promising diagnostic and prognostic biomarkers for GC (58). In our study, the expression of SMARCA4 in gastric cancer cells was higher than that in normal cells. OS analysis indicated that high expression of SMARCA4 was linked to poor prognosis, which is consistent with previous studies, and further confirmed our results. When SMARCA4 is mutated in gastric cancer, our data indicated that SMARCA4 does not act as a tumor suppressor, which may be due to the pathological activity of abnormal residual complexes of SWI/SNF.

Finally, we analyzed the protein expression of six key genes. We observed that the expression of TP53, AKT1, HRAS, PTEN, PIK3CA, SMARCA4 and BRCA1 in gastric cancer cells was higher than that in normal gastric cells. This is consistent with the results of our bioinformatics analysis. In genetic analysis, SNP is widely used as a kind of genetic markers, and some SNP located in genes may directly affect the protein structure or expression level. The six screened genes are highly expressed in gastric cancer cells, suggesting that these SNP mutant genes may play a role as oncogenes in gastric cancer. Based on this finding, a more in-depth study of the mechanism of these genes will help to reveal the role of SNP in the mechanism of cancer.

## Conclusion

In this study, through bioinformatics and experimental analyses, we found that six SNP-containing genes (TP53, AKT1, HRAS, PTEN, PIK3CA, SMARCA4 and BRCA1) may be key factors in the occurrence and prognosis of gastric cancer and participate in many pathways related to cancer development. Therefore, on this basis, further studies should be performed to detect the polymorphic sites of these genes and explore their corresponding expression levels, which can be used to predict the prognosis of patients. These findings will need to be verified in large-scale clinical studies to determine their accuracy and sensitivity in tumorigenesis and to guide the individualized treatment of patients. However, the focus of this study is to provide new ideas for clinical diagnosis and prognostic evaluation through bioinformatics analysis. Our results provide an important bioinformatics basis and related theoretical basis for guiding follow-up research on gastric cancer.

## Data Availability Statement

Publicly available datasets were used in this study. These can be found at The TCGA genomic data sharing platform (https://gdc.cancer.gov/).

## Ethics Statement

Written informed consent was obtained from the individual(s) for the publication of any potentially identifiable images or data included in this article.

## Author Contributions

HL and JG analyzed the data and wrote the manuscript. GC, YW, and SL assisted in editing the manuscript. YQ, GW, and RX contributed to the design of the study. WSQ and WWQ are the corresponding authors of the paper. All authors contributed to the article and approved the submitted version.

## Funding

This study was supported by two grants: Natural Science Foundation of China (81602068) and Beijing Xisike Clinical Oncology Research Foundation (Y-HR2018-185).

## Conflict of Interest

The authors declare that the research was conducted in the absence of any commercial or financial relationships that could be construed as a potential conflict of interest.
