# Supplementary material for: Identification and Validation of SNP-Containing Genes With Prognostic Value in Gastric Cancer via Integrated Bioinformatics Analysis
Source: Front Oncol. 2021 Apr 27;11:564296. doi: 10.3389/fonc.2021.564296 (PMC8112818; doi:10.3389/fonc.2021.564296)
Supplement: Supplementary file 1 [file Table_1.docx]

| Variables GC patients（N=381） | |
| --- | --- |
| Gender( Male/female) | 235/146 |
| Age(years, Mean±SD) | 63.47±10.25 |
| Tumor | |
| T1 | 25 |
| T2 | 93 |
| T3 | 172 |
| T4 | 91 |
| Regional lymph node | |
| N0 | 120 |
| N1 | 109 |
| N2 | 80 |
| N3 | 72 |
| Metastasis | |
| M0 | 349 |
| M1 | 32 |
| Histologic grade | |
| 1 | 24 |
| 2 | 138 |
| 3 | 219 |
| Pathologic stage | |
| 1 | 65 |
| 2 | 137 |
| 3 | 146 |
| 4 | 33 |

**Supplementary Table 1** **Basic characteristics of 381 GC patients.**
